# Transanal total mesorectal excision for a large leiomyosarcoma at the lower rectum: a case report and literature review

**DOI:** 10.1186/s40792-017-0289-z

**Published:** 2017-01-13

**Authors:** Nobuaki Hoshino, Koya Hida, Kenji Kawada, Takaki Sakurai, Yoshiharu Sakai

**Affiliations:** 1 Department of Surgery, Kyoto University Graduate School of Medicine, 54 Shogoin-Kawahara-cho, Sakyo-ku, Kyoto 606-8507 Japan; 2 Department of Diagnostic Pathology, Kyoto University Graduate School of Medicine, 54 Shogoin-Kawahara-cho, Sakyo-ku, Kyoto 606-8507 Japan

**Keywords:** Leiomyosarcoma, Rectum, Transanal total mesorectal excision, Case report

## Abstract

**Background:**

Rectal leiomyosarcoma (LMS) is an extremely rare disease. Previously, LMS was not properly distinguishable from gastrointestinal stromal tumor (GIST) until c-kit, a characteristic marker of GIST, was discovered in 1998. No standard therapeutic strategy for gastrointestinal LMS has been established except for surgical resection because of its rarity. Rectal LMS is often accompanied by symptoms, which can enable detection at a small size. However, when a large LMS is detected at the lower rectum, it is difficult to excise due to the narrow pelvic space.

**Case presentation:**

We present the case of an 86-year-old man with a large LMS. The LMS was asymptomatic and incidentally found at the lower rectum when he visited another hospital for management of benign prostatic hypertrophy. An abdominoperineal resection of the rectum was performed with combined resection of both seminal vesicles and a part of the prostate because tumor invasion was suspected. We used the hybrid method of laparoscopic and transanal total mesorectal excision (TaTME) approaches to achieve negative surgical margins. Late-onset urethral injury occurred in the postoperative course, which was successfully treated with a urethral catheter. The patient was discharged and received no adjuvant therapy. Local recurrence did not occur, but multiple lung metastases were detected 4 months later and the patient died 12 months after the surgery.

**Conclusions:**

This is the first report of the hybrid method of laparoscopic and TaTME approaches to remove a large LMS at the lower rectum.

## Background

Gastrointestinal leiomyosarcoma (LMS) is a rare entity that could not be accurately diagnosed until c-kit, a characteristic marker of gastrointestinal stromal tumor (GIST), was discovered in 1998 [1]. Rectal LMS is usually detected at a smaller size compared with colonic LMS because there are often symptoms in rectal LMS [2]. However, when a large tumor is found in the lower rectum, it is difficult to excise because of the narrow pelvic space. Transanal total mesorectal excision (TaTME) has been reported to be useful for removal of large pelvic tumors [3, 4]. Here, we present a case of large LMS in the lower rectum. The cavity of the lesser pelvis was almost completely occupied by the tumor. There has been no report about a large LMS in the lower rectum which was removed by the hybrid method of laparoscopic and TaTME approaches.

## Case presentation

**Fig. 1:**
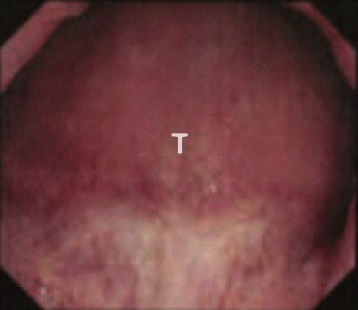
Endoscopic findings: colonoscopy revealed a large submucosal tumor at the lower rectum. *T* tumor

The patient was an 87-year-old man who had regularly visited another hospital for management of benign prostatic hyperplasia. His medical history included hypertension. In November 2013, a rectal mass was incidentally identified. Colonoscopy showed a large submucosal tumor at the lower rectum (Fig. 1). Pathological findings from biopsy specimen showed spindle cells arranged in irregular bands. Mitotic count was 3 per 10 high-power fields, and there was no tumor necrosis. Immunohistochemical staining for h-caldesmon, alfa-SMA, and desmin was positive, while staining for DOG-1, CD117 (c-kit), CD34, and S-100 was negative. Ki-67 index was 40%. The rectal mass was diagnosed as a rectal LMS. The tumor was very large and considered difficult to be removed by surgical intervention in that hospital. However, there were no other effective therapies except for surgical resection. In January 2014, he was therefore referred to our hospital for surgical treatment.

**Fig. 2:**
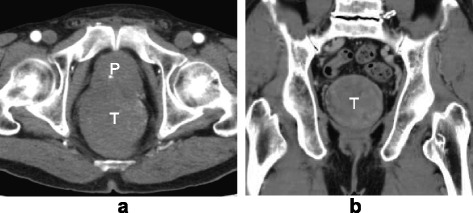
Contrast-enhanced computed tomography: **a** horizontal and **b** coronal sections. A large circle mass, 7.5 cm in diameter, was detected at the lower rectum. It occupied almost all space of the lesser pelvis. *P* prostate, *T* tumor

**Fig. 3:**
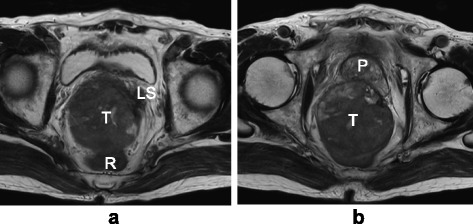
Magnetic resonance imaging T2-weighted images: Tumor invasion of the **a** left seminal vesicle and **b** prostate was suspected. *LS* left seminal vesicle, *P* prostate, *R* rectum, *T* tumor

Digital rectal examination revealed a large mass at the anterior wall of the lower rectum, and the inferior edge of the tumor was located 2 cm from the anal verge. Laboratory findings were within normal ranges. Contrast-enhanced computed tomography (CT) showed that the size of the tumor was 7.5 cm in diameter and that there were no signs of distant metastasis (Fig. 2). Abdominal magnetic resonance imaging (MRI) showed that the tumor originated from the anterior wall of the lower rectum and that it was suspected to have invaded the left seminal vesicle and prostate (Fig. 3). The tumor was close to the urinary bladder, but a cystoscope showed no signs of tumor invasion. ^18^F-fluorodeoxyglucose positron-emission tomography showed no distant metastasis.

**Fig. 4:**
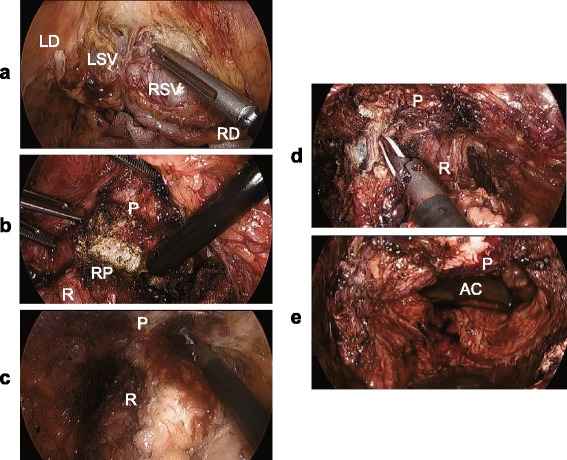
Intraoperative views: **a**, **b** Laparoscopic views and **c**–**e** transanal total mesorectal excision view. *AC* abdominal cavity, *LD* left seminal vesicle duct, *LSV* left seminal vesicle, *P* prostate, *R* rectum, *RD* right seminal vesicle duct, *RP* resected part of the prostate, *RSV* right seminal vesicle

**Fig. 5:**
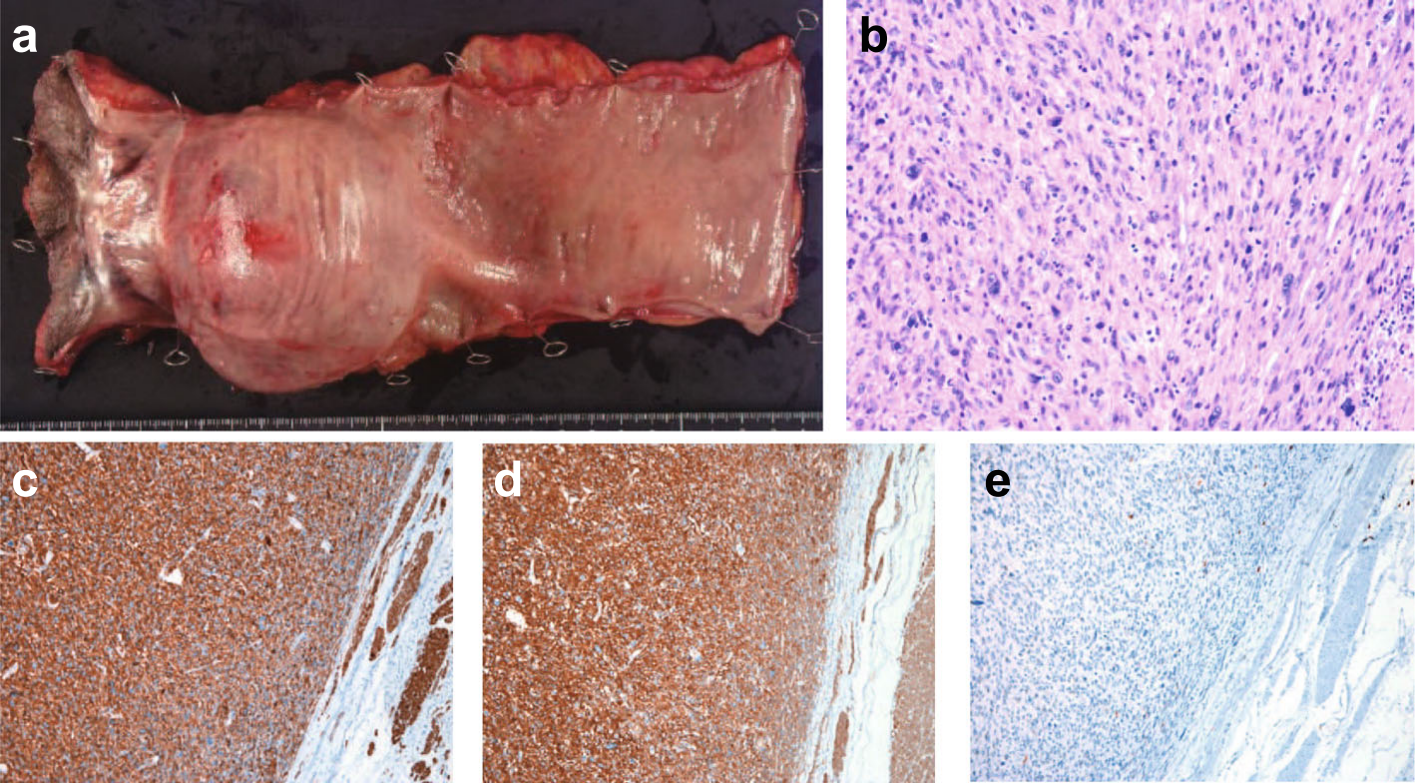
Surgical specimen and microscopic findings: **a** submucosal tumor at the lower rectum. **b** Hematoxylin-eosin stain (*20) revealed that spindle cells were arranged in irregular bands. Immunohistochemical staining was positive for calponin (**c**) and alfa-SMA (**d**), and negative for c-kit (**e**)

We made a diagnosis of a rectal LMS with suspected invasion of the left seminal vesicle and prostate. In March 2014, we performed an abdominoperineal resection of the rectum combined with both seminal vesicles and part of the prostate. We used the hybrid method of laparoscopic and TaTME approaches for better surgical view because it was apparently difficult to achieve negative surgical margins due to the large tumor located within a narrow pelvic space (Fig. 4). In this method, we used five trocars for laparoscopic surgery and a multiple access port for TaTME. A 12-mm trocar was inserted through an umbilical incision, and then the pneumoperitoneum was created. Another 12-mm trocar was placed in the right lower abdominal region, and three 5-mm trocars were inserted in the left lower abdominal and bilateral lateral regions. Pathological findings from the resected specimen showed that the tumor was a pleomorphic LMS and that tumor cells had invaded the left seminal vesicle and prostate with negative surgical margin. No lymph node metastasis was found. Immunohistochemical staining for calponin, alfa-SMA, and desmin was positive, while staining for DOG-1, c-kit, CD34, and S-100 was negative (Fig. 5). Tumor necrosis was found in the surgical specimen. The LMS was classified as grade 2 in Federation Nationale des Centres de Lutte le Cancer (FNCLCC) grading and stage IIb in TNM classification. In the postoperative course, the patient suffered from dysuria and needed intermittent self-catheterization. Late-onset urethral injury occurred 30 days after the operation, which was successfully treated with a urethral catheter. The patient was discharged 42 days after the operation. The patient received no adjuvant therapies such as chemotherapy and radiotherapy. Four months later, multiple lung metastases were detected on CT, although local recurrence was not found. The patient died 12 months after the operation.

### Discussion

It had been difficult to distinguish gastrointestinal LMS from GIST until c-kit was discovered in 1998 [1]. That is, the previously diagnosed gastrointestinal LMS is considered to include what is currently termed GIST [5]. According to the recent literature, the incidence of LMS was reported to be 1/50 to 1/65 of the incidence of GIST [2, 5]. LMS originates from smooth muscle cells and can arise anywhere in human body, although the main locations of LMS are the retroperitoneum, lower extremities, and the uterus [6]. Therefore, gastrointestinal LMS is very rare and its characteristics are still unclear.

To our knowledge, there are only 10 cases, including our case, in the English-language literature since 1998 in which immunohistochemical findings have been reported [2, 5, 7] (Table 1). The patients were three men and seven women. Median age was 65 years, ranging from 24 to 88. Average tumor size was 3.8 cm. Polypectomy was performed in two cases, anterior resection in three cases, abdominoperineal resection in four cases, and the surgical method was not reported in one case. Common characteristics of three recurrent cases were non-polypoid appearances such as Borrmann type 2- and plaque-like lesions. Our case had the largest tumor size, and there has been no report in which a large LMS in the lower rectum was removed by the hybrid method of laparoscopic and TaTME approaches.

**Table 1:** Reported cases of rectal leiomyosarcoma

Year	Author	Age (years)	Sex	Tumor size	Gross appearance	Mitotic count (/50HPF)	Recurrence	Outcome	Survival (months)
2001	Miettinen et al.	63	F	2.0	Intraluminal polyp	68	–	Alive	191
		32	F	2.3	Intraluminal polyp	>100	–	Alive	39
		79	F	2.5	Intraluminal polyp	26	–	Death from OD	55
		67	F	3.0	Plaque-like	>100	Peritoneum	Death from OD	61
		52	M	3.0	Intramural	46	–	Death from OD	70
		40	M	3.5	Intraluminal polyp	45	–	Alive	325
		24	F	5.5	Intraluminal polyp	62	–	NR	NR
		73	F	NR	NR	>100	–	Death from LMS	24
2013	Yamamoto et al.	88	F	6.5	Borrmann type 2-like	38	Skin, liver, peritoneum	Death from LMS	24
2016	Our case	87	M	7.5	Submucosal tumor	15	Lung	Death from LMS	12

*F* female, *HPF* high-power fields, *LMS* leiomyosarcoma, *M* male, *NR* not reported, *OD* other diseases

FNCLCC grading includes three factors; tumor differentiation, mitotic count, and tumor necrosis [8]. The Union for International Cancer Control/the American Joint Committee on Cancer staging system for soft tissue sarcoma assesses the following factors; tumor size, tumor depth, lymph node metastasis, distant metastasis, and histological grade [9]. However, the characteristics of gastrointestinal LMS have not been fully clarified because of its rarity [6]. Yamamoto et al. [5] reported that tumor-specific overall survival rate was 51.6% and that tumor size (≥5 cm) was significantly associated with a poor prognosis. They also suggested that tumor depth and necrotic area might be associated with patient survival. In our case, tumor size and gross appearance could be associated with a poor prognosis, while mitotic count was not. We surmised that patients with at least one risk factor might have a poor prognosis.

There has been no standard therapeutic strategy for gastrointestinal LMS. The effect of chemotherapy or radiotherapy is still unclear, whereas some antitumor drugs such as doxorubicin, ifosfamide, and eribulin are suggested to be effective for soft tissue sarcomas [10–12]. Surgical resection plays a critical role in the treatment for gastrointestinal LMS [6]. Despite the fact that lymph node metastasis is uncommon in soft tissue sarcoma, lymph node dissection seems to be necessary for gastrointestinal LMS because lymph node metastasis has been reported [5].

In our case, we performed abdominoperineal resection using the hybrid method of laparoscopic and TaTME approaches. It was considered difficult that the elderly patient underwent a total pelvic exenteration surgery and took care of a urostomy together with a colostomy. We assume that the TaTME approach is useful for the resection of a large pelvic tumor. Surgical margins were negative for tumor cells by this approach, but it was very close (<1 mm). There have been some reports on the effectiveness of radiotherapy against local recurrence [6, 13, 14], while radiotherapy was reported to be a risk factor for LMS [15]. Adjuvant radiotherapy or chemotherapy was not performed because there is no established adjuvant therapy for rectal LMS and the elderly patient did not hope to receive adjuvant therapies. However, local recurrence was not found irrespective of the short surgical margins. The late-onset urethral injury occurred after surgery. We conceived that the self-catheterization might make a negative impact on the occurrence. Four months after the surgery, multiple lung metastases were found. There have been some reports on the efficacy of chemotherapy in other LMS cases [6, 10, 12, 16, 17]. It also might prevent the recurrence of rectal LMS and contribute to prolong patient survival.

## Conclusions

We reported a case of LMS in the lower rectum. It was successfully removed by the hybrid method of laparoscopic and TaTME approaches. This method can be useful to achieve negative surgical margins in the resection of large pelvic tumors.

## Abbreviations

CT: Computed tomography
FNCLCC: Federation Nationale des Centres de Lutte le Cancer
GIST: Gastrointestinal stromal tumor
LMS: Leiomyosarcoma
MRI: Magnetic resonance imaging
TaTME: Transanal total mesorectal excision

## References

[1] Hirota S, Isozaki K, Moriyama Y, Hashimoto K, Nishida T, Ishiguro S (1998). Gain-of-function mutations of c-kit in human gastrointestinal stromal tumors. Science.

[2] Aggarwal G, Sharma S, Zheng M, Reid MD, Crosby JH, Chamberlain SM (2012). Primary leiomyosarcomas of the gastrointestinal tract in the post-gastrointestinal stromal tumor era. Ann Diagn Pathol.

[3] Hasegawa S, Okada T, Hida K, Kawada K, Sakai Y (2016). Transperineal minimally invasive approach for extralevator abdominoperineal excision. Surg Endosc..

[4] Wachter N, Worns MA, Dos Santos DP, Lang H, Huber T, Kneist W (2016). Transanal minimally invasive surgery (TAMIS) approach for large juxta-anal gastrointestinal stromal tumour. J Minim Access Surg.

[5] Yamamoto H, Handa M, Tobo T, Setsu N, Fujita K, Oshiro Y (2013). Clinicopathological features of primary leiomyosarcoma of the gastrointestinal tract following recognition of gastrointestinal stromal tumours. Histopathology.

[6] Duffaud F, Ray-Coquard I, Salas S, Pautier P (2015). Recent advances in understanding and managing leiomyosarcomas. F1000Prime Rep.

[7] Miettinen M, Furlong M, Sarlomo-Rikala M, Burke A, Sobin LH, Lasota J (2001). Gastrointestinal stromal tumors, intramural leiomyomas, and leiomyosarcomas in the rectum and anus: a clinicopathologic, immunohistochemical, and molecular genetic study of 144 cases. Am J Surg Pathol.

[8] Coindre JM, Trojani M, Contesso G, David M, Rouesse J, Bui NB (1986). Reproducibility of a histopathologic grading system for adult soft tissue sarcoma. Cancer.

[9] Edge SB, Compton CC (2010). The American Joint Committee on Cancer: the 7th edition of the AJCC cancer staging manual and the future of TNM. Ann Surg Oncol.

[10] Pautier P, Floquet A, Chevreau C, Penel N, Guillemet C, Delcambre C (2015). Trabectedin in combination with doxorubicin for first-line treatment of advanced uterine or soft-tissue leiomyosarcoma (LMS-02): a non-randomised, multicentre, phase 2 trial. Lancet Oncol.

[11] Judson I, Verweij J, Gelderblom H, Hartmann JT, Schoffski P, Blay JY (2014). Doxorubicin alone versus intensified doxorubicin plus ifosfamide for first-line treatment of advanced or metastatic soft-tissue sarcoma: a randomised controlled phase 3 trial. Lancet Oncol.

[12] Schoffski P, Chawla S, Maki RG, Italiano A, Gelderblom H, Choy E (2016). Eribulin versus dacarbazine in previously treated patients with advanced liposarcoma or leiomyosarcoma: a randomised, open-label, multicentre, phase 3 trial. Lancet.

[13] Tuan J, Vitolo V, Vischioni B, Iannalfi A, Fiore MR, Fossati P (2014). Radiation therapy for retroperitoneal sarcoma. Radiol Med.

[14] Roeder F, Ulrich A, Habl G, Uhl M, Saleh-Ebrahimi L, Huber PE (2014). Clinical phase I/II trial to investigate preoperative dose-escalated intensity-modulated radiation therapy (IMRT) and intraoperative radiation therapy (IORT) in patients with retroperitoneal soft tissue sarcoma: interim analysis. BMC Cancer.

[15] Horiguchi H, Takada K, Kamihara Y, Ibata S, Iyama S, Sato T (2014). Radiation-induced leiomyosarcoma of the prostate after brachytherapy for prostatic adenocarcinoma. Case Rep Oncol.

[16] Sagara K, Takayoshi K, Kusumoto E, Uchino K, Matsumura T, Kusaba H (2014). Favorable control of rapidly progressive retroperitoneal pleomorphic leiomyosarcoma with multimodality therapy: a case report. BMC Res Notes.

[17] Kasper B, Ouali M, van Glabbeke M, Blay JY, Bramwell VH, Woll PJ (2013). Prognostic factors in adolescents and young adults (AYA) with high risk soft tissue sarcoma (STS) treated by adjuvant chemotherapy: a study based on pooled European Organisation for Research and Treatment of Cancer (EORTC) clinical trials 62771 and 62931. Eur J Cancer.

